# Transcriptomic Analysis Suggests a Coordinated Regulation of Carotenoid Metabolism in Ripening Chili Pepper (*Capsicum annuum* var. *conoides*) Fruits

**DOI:** 10.3390/antiox11112245

**Published:** 2022-11-14

**Authors:** Shuyan Song, Shu-Yuan Song, Peiwen Nian, Dexin Lv, Yunhe Jing, Shan Lu, Qiang Wang, Fei Zhou

**Affiliations:** 1 State Key Laboratory of Pharmaceutical Biotechnology, School of Life Sciences, Nanjing University, Nanjing 210023, China; 2 Shenzhen Research Institute of Nanjing University, Shenzhen 518000, China; 3 State Key Laboratory of Tea Plant Biology and Utilization and International Joint Laboratory on Tea Chemistry and Health Effects, Anhui Agricultural University, Hefei 230036, China

**Keywords:** *Capsicum annuum*, carotenoid, chili pepper, fruit, ripen, transcriptome, transcription factor

## Abstract

Carotenoids are not only photosynthetic and photoprotective pigments in plants, but also essential antioxidative nutrients for human health. The fruit is the main plant organ that synthesizes and sequestrates carotenoids. Fruit ripening is a complicated developmental process, during which the rewiring of the metabolic network is tightly coordinated with the re-organization of cellular and organellular structures. Chili pepper (*Capsicum annuum*) is one of the major crops that accumulates a distinct level of carotenoids, especially capsanthin, in their ripened fruits. To elucidate how different metabolic and developmental scenarios are regulated in ripening chili pepper fruits, we analyzed the carotenoid profiles and transcriptomes of fruits at different ripening stages. Our pigment analysis indicated an opposite correlation between the contents of carotenoid species with β,β-structures (e.g., β-carotene, zeaxanthin, and capsanthin) and of lutein with the β,ε-structure, whereas lutein displayed a high correlation with chlorophylls during ripening. From the chili pepper *Zunla-1* genome, a full repertoire of 38 homologous genes encoding enzymes in the carotenoid biosynthetic pathway was identified. The fluctuations in their transcript abundances during ripening suggested different involvement of these genes in the regulation of carotenoid biosynthesis. We further searched genes of which the expression showed high correlations with the accumulation of β-carotene during the ripening process. Moreover, from the transcriptomic analysis, a total of 17 transcription factors that co-expressed with different groups of carotenoid biosynthetic genes were identified.

## 1. Introduction

Carotenoids are not only photosynthetic and photoprotective pigments in plants, but also essential antioxidative nutrients for human health. The fruit is the main plant organ that synthesizes and sequestrates carotenoids. Fruit ripening is a complicated developmental process during which a large number of metabolic and developmental processes are being re-wired and cell and organelle structures are also greatly changing [1,2,3]. During ripening, plastids in fruit cells showed drastic changes, which generally transform from chloroplasts into chromoplasts, accompanied by the degradation of chlorophylls and accumulation of carotenoids, together with the disassembly of the thylakoid membranes and assembly of carotenoid-sequestration structures, such as plastoglobules [3,4]. The metabolic pathways of both chlorophylls and carotenoids have been elucidated in a large number of organisms [5,6], and their fluctuations in ripening fruits have also been well-documented [7,8]. Different transcript factors, such as UV-damaged DNA-binding protein 1 (DDB1), DE-ETIOLATED1 (DET1), MADS6, R2R3-MYB, and Golden 2-like (GLK2), have been identified in various fruits as well, showing their specific regulatory impacts on pigment metabolism in ripening fruits [8,9,10,11,12]. However, it is still largely unclear how different metabolic and developmental scenarios are integrated during the ripening process.

**Figure 1:**
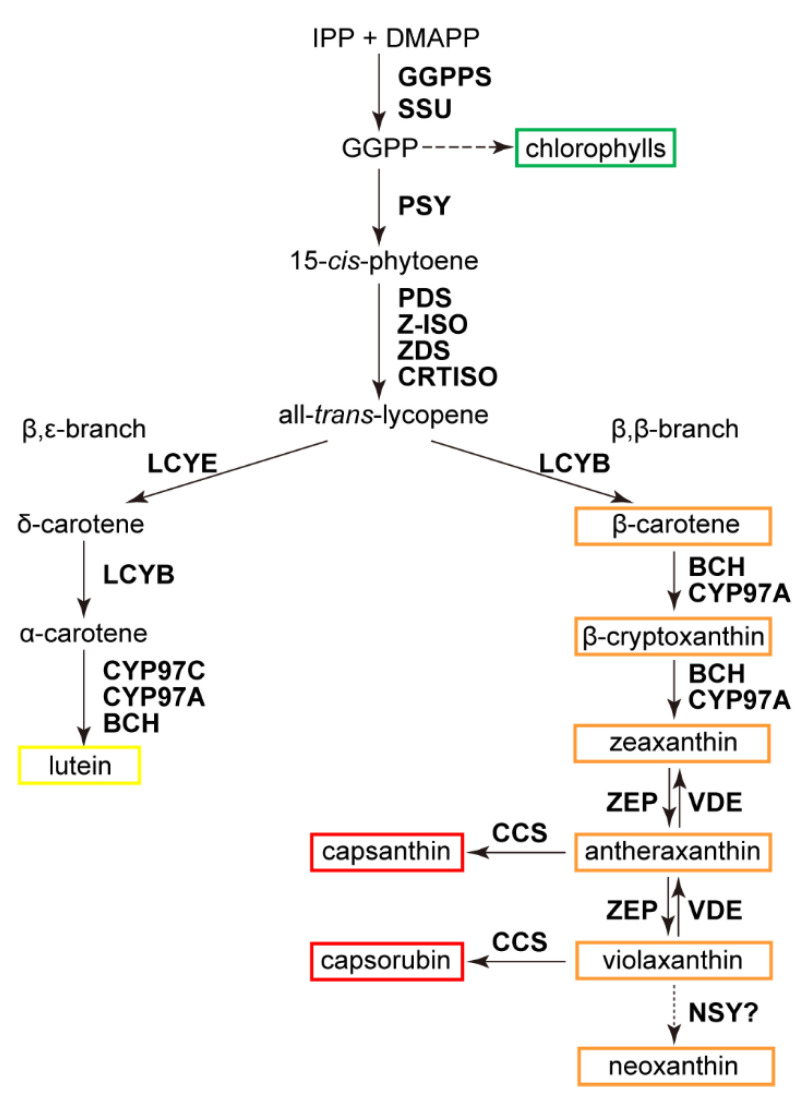
Carotenoid biosynthetic pathway. Enzymes are in bold face. Abbreviations for enzymes and substrates are: BCH, β-carotene hydroxylase; CCS, capsanthin/capsorubin synthase; CRTISO, carotene isomerase; CYP, cytochrome P450; DMAPP, dimethylallyl diphosphate; GGPP, geranylgeranyl diphosphate; GGPPS, GGPP synthase; IPP, isopentenyl diphosphate; LCYB, lycopene β-cyclase; LCYE, lycopene ε-cyclase; NSY, neoxanthin synthase; PDS, phytoene desaturase; PSY, phytoene synthase; VDE, violaxanthin de-epoxidase; ZEP, zeaxanthin epoxidase; ZDS, ζ-carotene desaturase; and Z-ISO, ζ-carotene isomerase. Pigments analyzed in this study are indicated: the β,β-branch and β, ε-branch carotenoids are boxed in yellow and orange, respectively; capsanthin and capsorubin are boxed in red; and chlorophylls are boxed in green. Question mark and dashed arrow indicate that the conversion of violaxanthin to neoxanthin in chili pepper was catalyzed by a presumed NSY.

In addition to tomato fruit [13], chili pepper (*Capsicum annuum*) is also one of the major carotenoid-accumulating crops in the Solanaceae family, of which capsanthin (and capsorubin, in some varieties) is the predominant carotenoid species in ripened fruits [7]. Metabolically, carotenoid biosynthesis starts from geranylgeranyl diphosphate (GGPP), a C_20_ linear terpenoid intermediate that is also used for synthesizing the phytol side-chain of chlorophylls and tocopherols [5,14,15]. Two molecules of GGPP are condensed by phytoene synthase (PSY), which specifically directs the metabolic flux toward carotenoid biosynthesis [14]. After two steps of desaturation by phytoene desaturase (PDS) and ζ-carotene desaturase (ZDS), and two steps of isomerization catalyzed by ζ-carotene isomerase (Z-ISO) and carotene isomerase (CRTISO), phytoene is converted into lycopene, the first branching point in the carotenoid metabolic pathway (Figure 1). Two types of cyclase, lycopene β- and ε-cyclases (LCYB and LCYE, respectively), catalyze the production of β-carotene (β,β-carotene) and α-carotene (β,ε-carotene), thus forming the β,β- and β,ε-branches beyond lycopene (Figure 1). Hydrocarbon β- and α-carotenes are further hydroxylated into zeaxanthin and lutein, respectively. Zeaxanthin epoxidase (ZEP) and violaxanthin de-epoxidase (VDE) catalyze together a reversible reaction among zeaxanthin, antheraxanthin, and violaxanthin (Figure 1) [6,14]. These three oxygenated β,β-branch carotenoids form the xanthophyll cycle in leaves as an essential photoprotective mechanism, and antheraxanthin and violaxanthin are also precursors for the biosynthesis of capsanthin and capsorubin, respectively, in chili pepper fruits (Figure 1) [16,17,18,19].

Recently, we analyzed variations of the carotenoid profile of chili pepper fruits at different ripening stages [20,21]. A coordinated upregulation of *LCYB* and downregulation of *LCYE* was demonstrated to favor the metabolic flux into the β,β-branch, and the biosynthesis of capsanthin and capsorubin in chili pepper fruits [20]. This indicated a sophisticated manipulation of the two carotenoid metabolic branches in ripening fruits. Here, we reported our analysis of transcriptomes of chili pepper fruits at different ripening stages. Our results clearly showed a synchronized regulation of carotenoid metabolism. Different proteins showing a high correlation with carotenoid metabolism during ripening, including transcription factors, were identified from our analysis.

## 2. Materials and Methods

### 2.1. Plant Materials and Growth Condition

**Figure 2:**
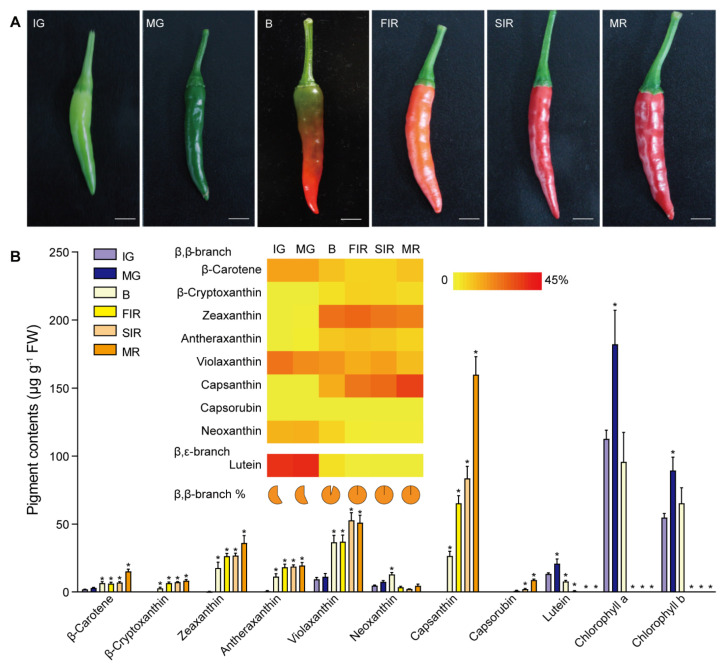
Variations of pigment contents in ripening chili pepper fruits. (**A**) Six stages of ripening chili pepper fruits. Abbreviations for each stage of samples: IG, immature green (*ca.* 15 days post anthesis [DPA]); MG, mature green (*ca.* 30 DPA); B, breaking (*ca*. 35 DPA); FIR, first immature red (*ca*. 38 DPA); SIR, second immature red (*ca.* 43 DPA); and MR, mature red (*ca*. 50 DPA). Scale bars = 1 cm. (**B**) Analyses of pigment contents in ripening chili pepper fruits. Carotenoids and chlorophylls were extracted from fruits at different ripening stages, separated by HPLC and then quantified. FW, fresh weight. Data are means ± SEM (*n* = 3). * Significantly different from the pigment content in IG stage (*p* < 0.05; one-way ANOVA and Tukey’s multiple comparisons test); the detailed statistics are provided in Table S2. Inset shows the percentages of separate carotenoid species in the total carotenoid pool. The percentage of all carotenoids of the β,β-branch in the pool is also calculated.

Seeds of red pepper (*Capsicum annuum* var. *conoides*) cultivar *Zunla-1*, of which the full genome has been sequenced [22], were purchased from Duoyouqi Technology Trading (Beijing, China). Plants were grown at 28 ℃ under a 16/8 h light/dark cycle. According to the pigment alterations during ripening, the ripening of pepper fruits was divided into six stages (Figure 2A), including immature green (IG, ca. 15 days post anthesis [DPA]), mature green (MG, ca. 30 DPA), breaker (B, ca. 35 DPA), first immature red (FIR, ca. 38 DPA), second immature red (SIR, ca. 43 DPA), and mature red (MR, ca. 50 DPA) [21]. Fruit samples from the six ripening stages with three biological replicates were prepared, using whole fruits (pericarp, placenta, and seeds). Samples were ground in liquid nitrogen into a fine powder, and then either immediately used for pigment extraction and RNA isolation, or stored at –80°C for future use.

### 2.2. Pigment Extraction and HPLC Analysis

Pigment extraction and high-performance liquid chromatography (HPLC) analysis were carried out as described by Wang et al. [21]. In brief, around 100 mg of well-homogenized pepper fruit powder were extracted in 1 mL of chloroform; at the same time, 5 μL of trans-*β*-apo-8′-carotenal (Sigma-Aldrich, 1 mg mL^−1^ dissolved in ethyl acetate) was added as an internal standard for relative quantification. Water (800 μL) was added and thoroughly mixed. After centrifugation at 5000× g for 5 min, the upper water phase was discarded and the organic phase was transferred into a new tube and air-dried under a nitrogen stream. The dried pigments were re-dissolved in 500 μL of isopropanol with the addition of 100 μL of methanolic KOH. After incubation at 4 ℃ overnight, 500 μL of water and 400 μL of chloroform were added and vortexed. Pigments were extracted into the organic phase by centrifugation at 16,000× g for 1 min. The pigments were dried and dissolved in 50 μL of ethyl acetate for HPLC analysis. A Waters 2695 separation module equipped with a 2998 photodiode array detector (PDA) and a Spherisorb ODS2 column (5 μm, 4.6 × 250 mm) (Waters, Milford, MA, USA) was used. A procedure of a 37 min gradient of ethyl acetate (0–100%) in acetonitrile-water-triethylamine (9:1:0.01, *v*/*v*) at 30 ℃ was used.

### 2.3. cDNA Library Construction and Sequencing

A total of 18 samples mentioned above were sent to Novogene (China) for cDNA library construction and sequencing. Briefly, total RNA of each sample was extracted, and the quality and purity of RNA were evaluated. In brief, RNA purity was checked through electrophoresis and a NanoPhotometer spectrophotometer (IMPLEN, CA, USA); RNA concentration was measured using Qubit RNA Assay Kit in Qubit 2.0 Flurometer (Life Technologies, CA, USA); and RNA integrity was assessed using the RNA Nano 6000 Assay Kit of the Bioanalyzer 2100 system (Agilent Technologies, CA, USA). Equal amounts of RNA per sample were used for cDNA library construction using NEBNext Ultra™ RNA Library Prep Kit for Illumina (NEB, USA), following the manufacturer’s recommendations. Library quality was assessed on the Agilent Bioanalyzer 2100 system. The library preparations were sequenced on an Illumina Hiseq 2000 platform and 100 bp paired-end reads were generated. For quantifying the transcript abundances of selected genes, total RNA was isolated using the RNAiso reagent (TaKaRa, Shiga, Japan) and incubated with DNase I (TaKaRa) to avoid genomic DNA contamination, according to the manufacturer’s protocol, and cDNA was synthesized using the HiScript III RT SuperMix for qPCR (+gDNA wiper) (Vazyme, Nanjing, China), following the manufacturer’s instructions. Gene expression levels were quantified by quantitative real-time PCR (qRT-PCR) using ChamQ SYBR qPCR Master Mix (Vazyme) with a Thermal Cycler Dice Real-Time System TP800 (TaKaRa), following the manufacturers’ instructions. Gene expression values were calculated according to the previously described comparative *C*_T_ method [23]. For normalizing gene expressions, *β-tubulin* was used as a reference [20]. All primers used in this study are listed in Supplemental Table S1.

### 2.4. Sequence Annotation

The raw reads of sequencing were filtered by removing reads containing the adaptor, reads with N coverage more than 10% (N means uncertain nucleotides), and reads with low quality (more than 50% nucleotide signals with sequencing quality values less than 5). In general, a total of 202.08 Gb of clean data were obtained and used for downstream analysis in this study. The reference genome of *Zunla-1* (*C*. *annuum* L.) and gene annotation files were downloaded from NCBI (https://www.ncbi.nlm.nih.gov/bioproject/186921, accessed on 1 January 2017). The sequences were mapped to the reference genome using TopHat [22,24]. The expression levels of genes (reads per kilo bases per million reads, RPKM) were analyzed by HTSeq [25].

### 2.5. Analysis of Differentially Expressed Genes

To screen for differentially expressed genes in six ripening stages, the DESeq R package was used [26]. Genes with an adjusted *p* value < 0.05 by Benjamini and Hochberg’s approach were regarded as differentially expressed genes. The heat map of clustered analysis was displayed using the pheatmap package from R [27].

### 2.6. Correlation Analysis

The Pearson correlation coefficient of all gene transcript abundances (in RPKM) with fluctuations of β-carotene contents during ripening was calculated using the cor function in R, and the *p* value was measured by the cor.test function in R [28]. Genes with RPKM > 5 during the breaker stage with a significant correlation (*R*^2^ > 0.8, *p* value < 0.05) were screened as possible candidate genes related to β,β-branch carotenoid accumulation. Likewise, the Pearson correlation coefficient of all TFs with highly expressed carotenoid metabolism genes was separately measured by the cor function in R, and TFs showing significant correlation with multiple carotenoid metabolism genes were counted [28]. Correlation coefficients between paired pigments were also calculated through the cor function in R [28]. The principal components analysis (PCA) of pigments based on the changes in content from different stages was performed using the OmicShare tools with default settings (https://www.omicshare.com/tools, accessed on 27 July 2022).

## 3. Results and Discussion

### 3.1. Coordinated Fluctuations of Carotenoid Constituents in Ripening Fruits

Previously, we divided the ripening process of chili pepper into six different stages (Figure 2A), i.e., immature green (IG), mature green (MG), breaker (B), first immature red (FIR), second immature red (SIR), and mature red (MR), and quantified the carotenoid contents in fruits at each stage [20,21]. As it has been widely reported, capsanthin was significantly accumulated from the B stage throughout the ripening process (Figure 2B and Table S2). On the contrary, lutein, the only carotenoid constituent of the β,ε-branch identified in chili pepper fruits, accumulated at the IG and MG stages, but decreased to an undetectable level during the ripening, resembling the changes in chlorophylls *a* and *b* (Figure 2B and Table S2). For the three xanthophylls, our quantification showed a detectable level of violaxanthin, but not antheraxanthin and zeaxanthin, in green fruits (IG and MG), resembling their presence in leaves under normal growth light (Figure 2B and Table S2). However, from the B stage, increasing levels of all three xanthophylls were observed. Antheraxanthin and violaxanthin were immediate substrates for the biosynthesis of capsanthin and capsorubin [17,19]. With their accumulation, contents of both capsanthin and capsorubin also showed distinct increases from the B and FIR stages, respectively (Figure 2B and Table S2). Although violaxanthin is also a precursor for producing neoxanthin, which is a substrate for ABA biosynthesis, neoxanthin was only found to accumulate from the IG to B stages, and then declined to an undetectable level in MR fruits (Figure 2B and Table S2). Therefore, the content of neoxanthin did not fluctuate with other β,β-branch carotenoid species.

As the content of the entire carotenoid bouquet increased during ripening, it’s the net amount of each constituent did not reflect its corresponding share in the carotenoid pool. To this end, we calculated the percentage of every constituent in the carotenoid pool of fruits at each ripening stage (Figure 2B). From our calculations, zeaxanthin showed a distinct accumulation in the B and FIR stages, and was still the major carotenoid species in the next two stages, until capsanthin dominated at the final MR stage (Figure 2B). The percentage of antheraxanthin in the carotenoid pool also showed an increase at the B and FIR stages, agreeing with its function as the precursor for capsanthin biosynthesis (Figure 2B). In the β,ε-branch, although lutein was one of the major carotenoid species in green fruits (IG and MG stages), its net content and percentage of total carotenoids became almost zero from the breaker stage (Figure 2B).

**Figure 3:**
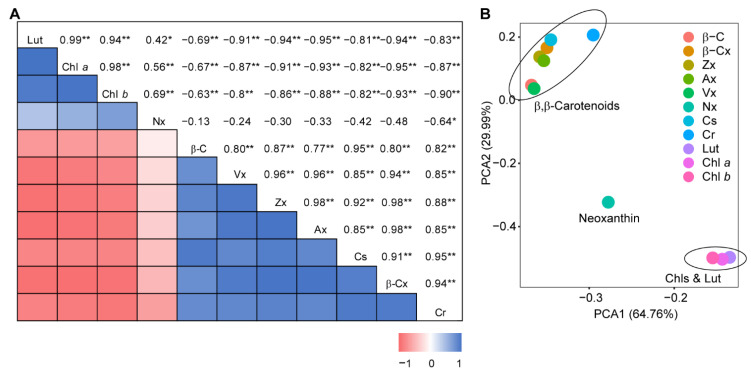
Correlation analysis of carotenoid constituents in chili pepper fruit. (**A**) The correlation matrix between carotenoid contents in pepper fruits. Asterisks indicate significant differences at *p* < 0.05 (*) and *p* < 0.01 (**) levels (measured by the cor.test function in R). (**B**) The principal components analysis (PCA) of pigment constituents during ripening. Abbreviations for pigments are β-C, β-carotene; β-Cx, β-cryptoxanthin; Zx, zeaxanthin; Ax, antheraxanthin; Vx, violaxanthin; Nx, neoxanthin; Cs, capsanthin; Cr, capsorubin; Lut, lutein; Chl *a*, chlorophyll *a*; Chl *b*, chlorophyll *b*.

We then performed a Pearson correlation analysis of the fluctuations in different carotenoids. Our results revealed a clear correlation among carotenoid species of the β,β-branch, except for neoxanthin (Figure 3). It is interesting that lutein of the β,ε-branch and chlorophyll *a* and *b* also demonstrated correlated variations during ripening (Figure 3). Taken together, our results indicated opposite correlations between the β,β-branch carotenoids and the pigment group of lutein and chlorophylls during the ripening process.

### 3.2. Concerted Expression of Carotenoid Metabolic Genes

To resolve the molecular mechanisms underlying the coordinated variations of different carotenoid species, we performed RNA-seq analysis to elucidate the global regulation at the transcriptional level. For each ripening stage, transcriptomes from three replicates were sequenced. Quality control analysis indicated that the sequencing quality was sufficient for carrying out further analysis (Tables S3 and S4). A total of 202.08 Gb reads were generated, from which 29,330 genes were successfully mapped to the chili pepper *Zunla-1* genome [22]. An overall cluster analysis of samples from six stages, based on differentially expressed genes, was carried out. According to the analysis, the samples subjected to IG and MG, B and FIR, and SIR and MR were clustered together, respectively. Among them, expression patterns of IG and MG showed the greatest differences from the other stages. Drastic transcriptional changes on a global level were observed, beginning with the B stage, which was consistent with carotenoid profiles (Figure S1 and Figure 2).

**Figure 4:**
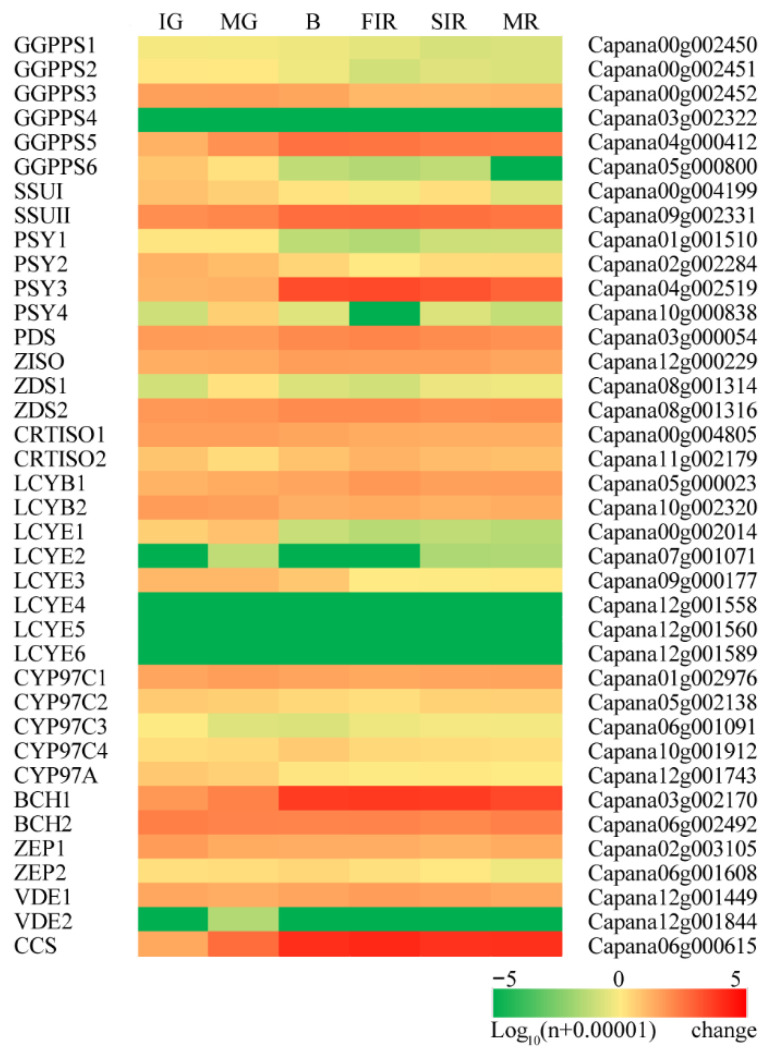
Expression levels of each of the genes for carotenoid metabolism at different ripening stages. B, breaker; FIR, first immature red; IG, immature green; MG, mature green; MR, mature red; and SIR, second immature red. Logarithms of the reads per kilobases per million reads (RPKM) value of each gene (plus 0.0001) are presented. Abbreviations for enzymes are BCH, β-carotene hydroxylase; CCS, capsanthin/capsorubin synthase; CRTISO, carotene isomerase; CYP, cytochrome P450; GGPPS, geranylgeranyl diphosphate synthase; LCYB, lycopene β-cyclase; LCYE, lycopene ε-cyclase; PDS, phytoene desaturase; PSY, phytoene synthase; SSU, small subunit homolog of GGPPS; VDE, violaxanthin de-epoxidase; ZEP, zeaxanthin epoxidase; ZDS, ζ-carotene desaturase; and Z-ISO, ζ-carotene isomerase. Accession numbers of all genes are provided in Table S5.

To further clarify the transcriptional pattern of genes involved in carotenoid biosynthesis, a total of 38 homologous sequences for enzymes in the entire pathway were identified by sequence blast against the chili pepper genome, from the biosynthesis of geranylgeranyl diphosphate (GGPP) to the production of capsanthin and capsorubin (Figure 1 and Table S5). For some of the homologs, their transcripts were either undetectable or at very low levels in all ripening stages, indicating that they were specifically expressed in other tissues/organs (Figure 4). We also picked a few genes from the list and determined their transcript abundances at different ripening stages by qRT-PCR. The fluctuations of these genes were identical with the RNA-seq results, which further confirmed the RNA-seq quality (Figure S2).

GGPP is a precursor shared by the biosynthesis of not only carotenoids but also the phytol side chain of chlorophylls [5,15,29]. GGPP synthases (GGPPSs), the enzymes that catalyze the biosynthesis of GGPP, also shared high sequence similarities with geranyl diphosphate synthase (GPPS) and two types of catalytically inactive small subunit proteins (SSUI and SSUII) [30]. Among the eight homologous genes identified from the genome, transcripts of *CaGGPPS4* were not detectable in any samples (Figure 4). *CaGGPPS1* and *CaGGPPS2* showed very low expression in the two green stages. Transcript abundances of both *CaGGPPS6* and *CaSSUI* declined from the IG stage, together with the decline of chlorophyll contents. The expression of *CaGGPPS3* increased at first, but then declined. The expression of *CaGGPPS5* and *CaSSUII* showed fluctuations similar to our previous report [21], which both peaked at the breaker stage, indicating their predominance (Figure 4).

Phytoene synthase (PSY) is the entry enzyme that directs the metabolic flux from GGPP into carotenoid biosynthesis [6]. Four *PSY* homologous genes were identified, among which *CaPSY3* showed an overwhelming expression at the breaker stage, and transcripts of both *CaPSY1* and *CaPSY4* were barely detectable (Figure 4). This was consistent with our previous report [21]. Phytoene desaturase (PDS) is regarded as a rate-limiting enzyme in the carotenoid biosynthetic pathway [31]. In the chili pepper, PDS is encoded by a single gene, *CaPDS*. Its transcript abundance increased from the breaker to FIR stages, but decreased afterward. Beyond PDS, the chili pepper has two homologous genes for ζ-carotene desaturase (ZDS). Only *CaZDS2* was found to be expressed in ripening fruits, and showed a fluctuation resembling that of *CaPDS* (Figure 4).

Lycopene is the branching point in the carotenoid metabolic pathway, from which lycopene β- and ε-cyclase (LCYB and LCYE, respectively) manipulate the metabolic flux into the β,β- and β,ε-branches [32]. Previously, we demonstrated the coordinated upregulation of *CaLCYB1* and downregulation of *CaLCYE3*, the two major *LCY* genes in ripening chili pepper fruits [20]. These results were confirmed by our RNA-seq data (Figure 4).

The hydroxylation of hydrocarbon carotenes of the two branches (β-carotene of the β,β-branch and α-carotene of the β,ε-branch) was catalyzed by non-heme β-carotene hydroxylases (BCHs) or cytochrome P450-type carotene β- and ε-hydroxylases (CHYB and CHYE, respectively) of the CYP97 family [6]. Two *BCH* and five CYP97 homologous genes were identified from the chili pepper genome. *CaBCH1* and *CaBCH2* had comparable transcript abundances in the two green stages, but that of *CaBCH1* showed significant upregulation at the breaker stage, whereas the expression level of *CaBCH2* was largely stable (Figure 4). Among the five *CYP97* members, one belonged to the *CYP97A* subfamily for carotene ε-hydroxylases (*Capana12g001743*), which only showed low expressions in the green stages. The other four members belonged to the *CYP97C* subfamily for carotene β-hydroxylases. Except for *CaCYP97C3*, which was not expressed in fruits, the other three genes showed similar expression patterns with *CaBCH2* (Figure 4).

Zeaxanthin epoxidase (ZEP) and violaxanthin de-epoxidase (VDE) catalyzed the reversible conversion among zeaxanthin, antheraxanthin, and violaxanthin [6]. Two homologous genes for both enzymes were identified from the chili pepper genome (Figure 1 and Table S5). Transcript abundance of *CaZEP1* was much higher than that of *CaZEP2*, and peaked at IG stage. The expression level of *CaVDE1* peaked at the FIR stage, whereas that of *CaVDE2* was very low in the fruits at different stages (Figure 4).

Capsanthin/capsorubin synthase (CCS) is the enzyme that catalyzes the biosynthesis of capsanthin and capsorubin from zeaxanthin and violaxanthin, respectively [17,19]. Our quantification demonstrated that the transcripts of *CaCCS* started to accumulate from the IG stage, peaked at the FIR stage, and maintained a relatively high level at the final MR stage (Figure 4).

### 3.3. Genes Co-Expressed with Carotenoid Metabolism in Ripening Fruits

Besides pigment metabolism, there are also other metabolic and developmental scenarios during the ripening process. To figure out whether other genes were also coordinated with carotenoid metabolism, we calculated the correlation between the expression levels of all genes and β-carotene content, which is the common precursor of all carotenoids in the β,β-branch, and directly reflects the metabolic flux into the β,β-branch. We screened all genes with abundances of RPKM > 5 in the transcriptomes. The top 25 genes that showed high correlations with β-carotene content (*R^2^* > 0.8, *p* < 0.05) were listed in Table 1, and their expression levels were presented in Figure S3. From the list, it is clear that the expression of genes related to desiccation and metabolism was highly correlated with carotenoid metabolism during the ripening process. Interestingly, genes for an oxygen-independent coproporphyrinogen-III oxidase-like protein, which may be involved in heme biosynthesis, and pheophorbide *a* oxygenase, which is an enzyme for chlorophyll breakdown, were both highly correlated with β-carotene fluctuations [5,33] (Table 1). To our surprise, no genes for enzymes or structural proteins related to carotenoid metabolism showed a high correlation with β-carotene fluctuations in our analysis (Table 1).

Moreover, we further analyzed the transcription factors (TFs) that may participate in the regulation of carotenoid metabolism during ripening. From the transcriptomes, we looked for TF genes that showed high correlations with at least four carotenoid metabolic genes. A total of 17 TFs were identified (Table 2 and Figure S4). From the list, *CaPSY2* and *CaLCYE3* shared their expression with a group of eight TF genes, which were either not correlated with, or negatively correlated with the other five enzyme genes. We termed these TFs β,ε-correlated TFs. For *CaPSY3*, *CaPDS*, *CaZISO*, and *CaBCH1*, eight TF genes showed identical correlations with at least three of these genes and *CaCCS*, and were named β,β-correlated TFs. A MYB family TF (*Capana12g002172*) showed positive correlations with *CaPSY3*, *CaPDS*, *CaZISO*, *CaBCH1*, and *CaCCS*, and a negative correlation with only CaPSY2 (Table 2). A NAC family TF (*Capana09g000936*) showed only positive correlations with *CaPSY3*, *CaBCH1*, and *CaCCS* (Table 2). These results strongly suggest that *CaPSY2* is likely co-regulated with *CaLCYE3*, which modulates the biosynthesis of lutein to fluctuate with chlorophylls during ripening, and also further support the high correlation among variations in different β,β-branch carotenoids toward capsanthin/capsorubin.

## 4. Conclusions

Overall, we analyzed the fluctuations of carotenoid constituents in chili pepper fruits at different ripening stages, which demonstrated opposite correlations between the β,β-branch carotenoids and the pigment group of lutein (β,ε-branch) and chlorophylls during the ripening process. Our transcriptome analysis revealed the differential expression patterns of a full repertoire of 38 carotenoid biosynthesis-related genes. Further correlation analysis identified different groups of TFs that may take part in the coordinated regulation of these two correlations during the ripening process of chili pepper fruits, which eventually resulted in an accumulation of capsanthin/capsorubin with diminished lutein and chlorophylls. Further functional characterization of the identified TFs would help to better understand the rewiring of the metabolic network, especially those for carotenoids and chlorophylls, in ripening fruits.

## Figures and Tables

**Table 1:** Genes highly associated with the β-carotene content in ripening fruits ^1^.

Gene ID	Annotation
*Capana08g001508*	Desiccation-related protein
*Capana03g001333*	Oxygen-independent coproporphyrinogen-III oxidase-like protein
*Capana11g000180*	Pheophorbide *a* oxygenase
*Capana04g000040*	Dihydroflavonol-4-reductase
*Capana02g002069*	Protein MOTHER of FT and TF 1
*Novel05452*	Metallothionein-like protein type 2
*Capana00g003749*	Bifunctional epoxide hydrolase 2
*Capana08g000991*	BAG family molecular chaperone regulator 6
*Capana08g001915*	Cruciferin PGCRURSE5
*Capana02g001627*	Protein SRG1
*Capana10g001975*	Zinc finger A20 and AN1 domain-containing stress-associated protein 4
*Capana01g004002*	Heat shock 70 kDa protein 7
*Capana01g002803*	Probable WRKY transcription factor 23
*Capana05g002410*	Receptor-like protein kinase HERK 1
*Capana03g003377*	Cytochrome P450 94A1
*Capana08g001008*	Desiccation protectant protein LEA14 homolog
*Capana00g002331*	Phosphopantetheine adenylyltransferase
*Capana10g000384*	Bifunctional epoxide hydrolase 2
*Capana08g001371*	Probable acyl-activating enzyme 6
*Capana08g000393*	Nuclear transcription factor Y subunit A-6
*Capana10g001621*	Transcription factor HBP-1b (c38)
*Capana02g001485*	Potassium channel KAT1
*Capana07g001105*	UDP-glycosyltransferase 85A1
*Capana04g000461*	Cytochrome P450 82A4
*Capana06g002950*	S-Norcoclaurine synthase

^1^ Top 25 substantially expressed (RPKM > 5 at the breaker stage) genes for which transcript abundances showed high correlations with fluctuations in β-carotene content during ripening (R^2^ > 0.8 and *p* < 0.05); detailed expression levels are presented in Figure S3. Gene sequences are accessible through the *Zunla-1* genome (https://solgenomics.net/tools/blast/, accessed on 1 January 2019).

**Table 2:** Transcription factors co-expressed with carotenoid biosynthetic genes in chili pepper fruits.

Type	Gene ID	Correlation ^1^						
		*CaPSY2*	*CaLCYE3*	*CaPSY3*	*CaPDS*	*CaZISO*	*CaBCH1*	*CaCCS*
β,ε-correlated TFs								
Dof	*Capana02g001972*	+	+	-	0	0	-	0
G2-like	*Capana12g002836*	+	+	-	0	0	-	0
bHLH	*Capana03g004251*	+	+	-	0	-	-	0
WRKY	*Capana03g003085*	+	+	0	0	-	-	-
ERF	*Capana04g001803*	+	+	0	0	0	-	-
MYB	*Capana06g002789*	+	+	0	0	0	-	-
B3	*Capana01g004070*	-	-	0	+	0	+	+
NF-YC	*Capana11g000539*	-	-	0	0	0	+	+
β,β-correlated TFs								
MYB	*Capana12g002172*	-	0	+	+	+	+	+
NAC	*Capana04g001537*	0	0	+	+	+	+	+
GRAS	*Capana07g001537*	0	0	+	+	+	+	+
MIKC	*Capana07g001940*	0	0	+	+	0	+	+
MYB	*Capana07g001604*	0	0	-	-	-	-	-
bHLH	*Capana08g001686*	0	0	-	-	-	-	-
ARF	*Capana11g000076*	+	0	-	0	-	-	-
GRF	*Capana01g000919*	+	0	-	0	-	-	-
NAC	*Capana09g000936*	0	0	+	0	0	+	+

^1^ The symbols “+”, “-”, and “0” indicate positive, negative, and no correlations, respectively, between the corresponding transcription factor and carotenoid metabolic gene at the *p* > 0.05 level. Gene sequences are accessible through the *Zunla-1* genome (https://solgenomics.net/tools/blast/, accessed on 1 January 2019).

## Data Availability

Data are contained within the article and Supplementary Material.

## Supplementary Materials

The following are available online at https://www.mdpi.com/article/10.3390/antiox11112245/s1. Table S1. Primers used in this study. Table S2. Statistical analysis of pigment contents in ripening chili pepper fruits. Table S3. RNA-seq data quality information. Table S4. Alignment of the RNA-seq reads and reference genome. Table S5. Genes for carotenoid biosynthesis enzymes identified from the chili pepper *Zunla-1* genome. Figure S1. Cluster analysis of differentially expressed genes (DEGs) among chili pepper fruit ripening stages. Figure S2. Transcript abundance of selected carotenoid biosynthesis genes in chili pepper fruits at different ripening stages. Figure S3. Transcript abundance of genes highly associated with β-carotene content in ripening fruits. Figure S4. Transcript abundance of transcription factors co-expressed with carotenoid biosynthetic genes in chili pepper fruits.
