# Supplementary material for: Transcriptomic Analysis Suggests a Coordinated Regulation of Carotenoid Metabolism in Ripening Chili Pepper (Capsicum annuum var. conoides) Fruits
Source: Antioxidants (Basel). 2022 Nov 14;11(11):2245. doi: 10.3390/antiox11112245 (PMC9686772; doi:10.3390/antiox11112245)
Supplement: Supplementary file 1 [file antioxidants-11-02245-s001.zip › antioxidants-2007314-supplementary.pdf]

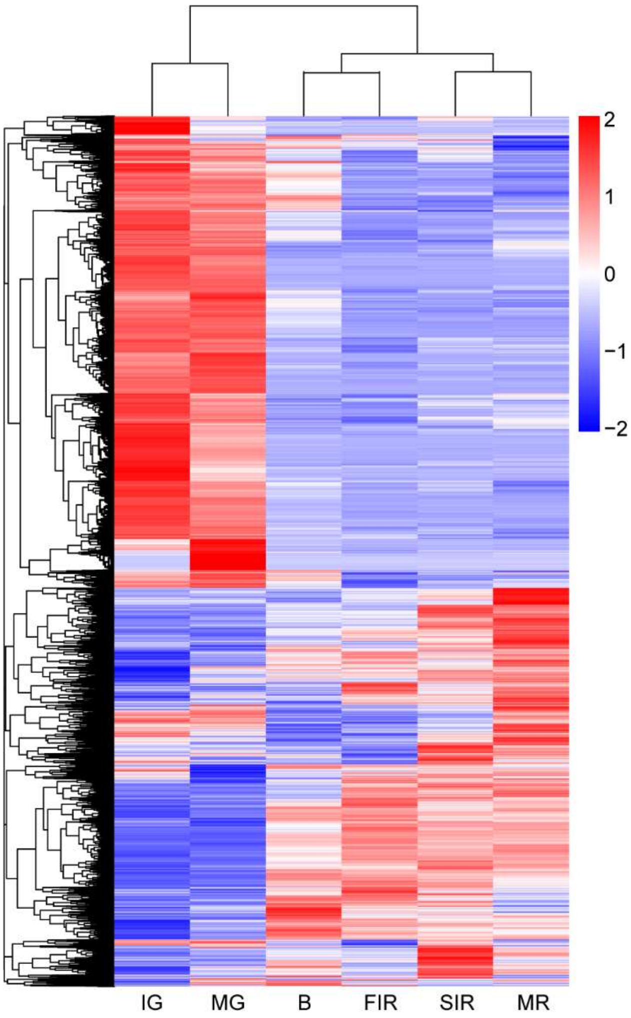

Figure S1. Cluster analysis of differentially expressed genes (DEGs) among chili pepper fruit ripening stages. Abbreviations for six ripening stages are: IG, immature green; MG, mature green; B, breaker; FIR, first immature red; SIR, second immature red; and MR, mature red. Logarithms of the reads per kilo bases per million reads (RPKM) value of each differentially expressed gene (plus 0.1) were calculated for hierarchical cluster analysis. Color bar indicates Z-score after normalization.

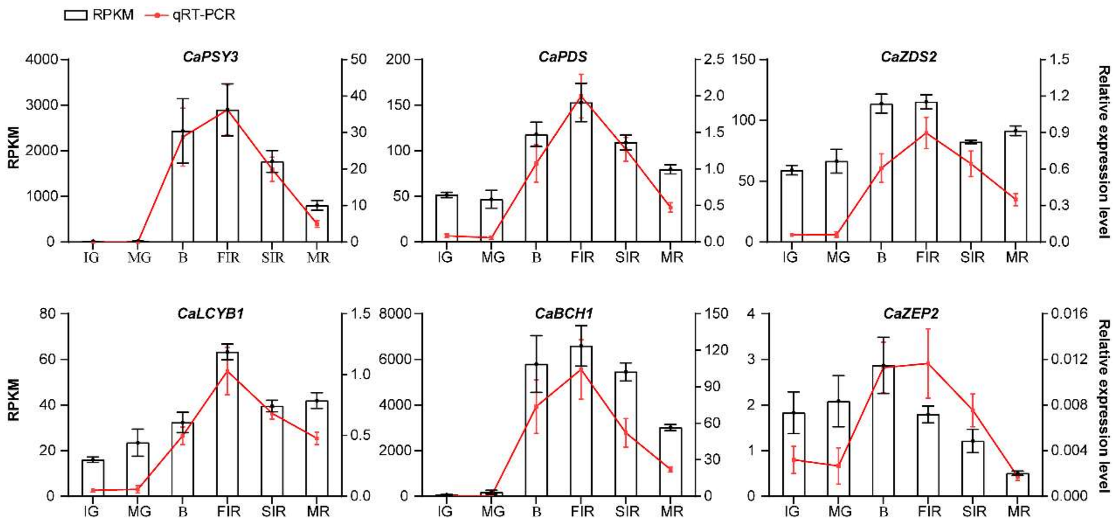

Figure S2. Transcript abundance of selected carotenoid biosynthesis genes in chili pepper fruits at different ripening stages. Transcript abundance of each gene determined by RNA-seq is presented as reads per kilo bases per million reads (RPKM), and by qRT-PCR is normalized against the expression level of *β*-tubulin. Different ripening stages are: IG, immature green; MG, mature green; B, breaker; FIR, first immature red; SIR, second immature red; and MR, mature red. Abbreviations for selected chili pepper (*Capsicum annuum* L., Ca) genes are: PSY, phytoene synthase; PDS, phytoene desaturase; ZDS, ζ-carotene desaturase; LCYB, lycopene β-cyclase; BCH, β-carotene hydroxylase; and ZEP, zeaxanthin epoxidase. Data represents means ± SEM (n = 3).

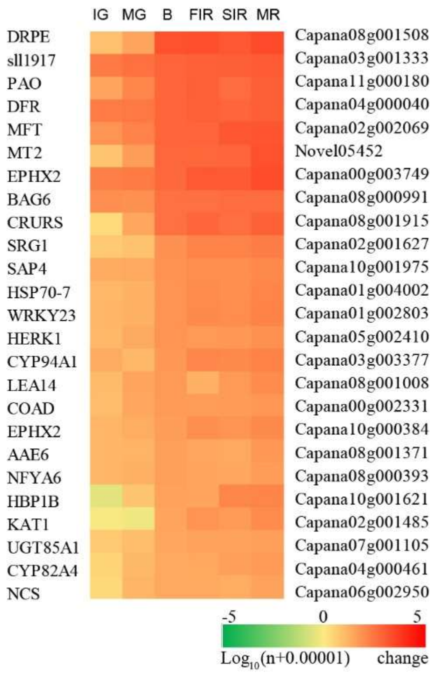

Figure S3. Transcript abundance of genes highly associated with β-carotene content in ripening fruits. Logarithms of the reads per kilo bases per million reads (RPKM) value of each gene (plus 0.0001) are presented. The full name for each gene can be found in Table 1.

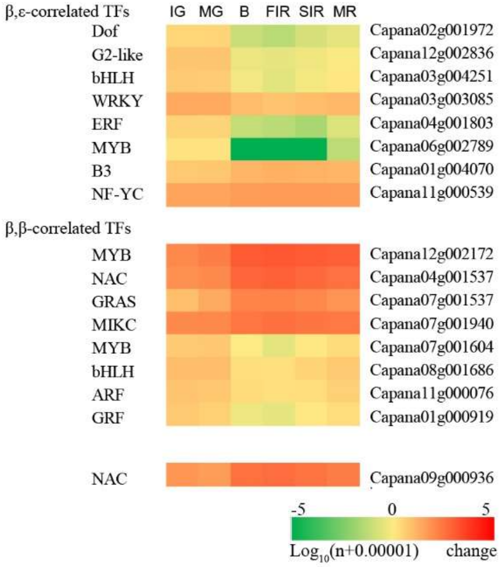

Figure S4. Transcript abundance of transcription factors co-expressed with carotenoid biosynthetic genes in chili pepper fruits. Logarithms of the reads per kilo bases per million reads (RPKM) value of each gene (plus 0.0001) are presented. The annotations for each gene are as follows: Dof, Dof zinc finger protein PBF; G2-like, transcription repressor KAN1; bHLH, transcription factor bHLH128; WRKY, probable WRKY transcription factor 20; ERF, ethylene-responsive transcription factor ERF021; MYB (Capana06g002789), transcription factor MYB59; B3, B3 domain-containing protein REM13; NF-YC, Dr1-associated corepressor; MYB (Capana12g002172), transcription factor MYB1R1; NAC (Capana04g001537), NAC domain-containing protein 2; GRAS, Scarecrow-like protein 32; MADS, floral homeotic protein AGAMOUS; MYB (Capana07g001604), Myb-related protein Myb4; bHLH (Capana08g001686), transcription factor PIF3; ARF, auxin response factor 18; GRF, growth-regulating factor; and NAC (Capana09g000936), NAC domain-containing protein.

Table S1. Primers used in this study.

Accession number	Name	Sequence
Capana04g000187	β-tubulin-qF	GAGGGTGAGTGAGCAGTTC
	β-tubulin-qR	CTTCATCGTCATCTGCTGTC
Capana04g002519	CaPSY3-qF	CTTGTGAGTAGTTGGGTTGGCA
	CaPSY3-qR	GCTTCGGTTACTGATGTTGTTG
Capana03g000054	CaPDS-qF	TTGGAGGCTGCATTCTTATC
	CaPDS-qR	CTCCAGCAGTATTGGTTTGT
Capana08g001316	CaZDS2-QF	AAATGGTCCAACGGGATTC
	CaZDS2-QR	GCAAGTCCAGCTCCAATAATA
Capana05g000023	CaLCYB1-qF	GAAGTCATTCGGGTGATGAG
	CaLCYB1-qR	CTTGTAGCCGGTAAGTCAAG
Capana03g002170	CaBCH1-QF	CCTCCTTGACTATGGTTTCTTC
	CaBCH1-QR	CATTAGCTACAGGTCCAACAG
Capana06g001608	CaZEP2-QF	GCAGTATTGTGCTGGTTGGC
	CaZEP2-QR	GCTTTGAACGCTTCCTCGAC

Table S2. Statistical analysis of pigment contents in ripening chili pepper fruits.

Pigments	Comparisons[1]	Significance[2]	Adjusted *P* Value	Fold change (log_2)[3]
β-Carotene	IG vs. MG	ns	0.7801	0.5940
	IG vs. B	***	0.0006	1.7564
	IG vs. FIR	**	0.0016	1.6463
	IG vs. SIR	***	0.0003	1.8312
	IG vs. MR	****	<0.0001	2.9489
β-Cryptoxanthin	IG vs. MG	ns	>0.9999	0.0000
	IG vs. B	**	0.0013	8.1115
	IG vs. FIR	****	<0.0001	9.3553
	IG vs. SIR	****	<0.0001	9.5063
	IG vs. MR	****	<0.0001	9.6841
Zeaxanthin	IG vs. MG	ns	>0.9999	4.5521
	IG vs. B	***	0.0001	10.7897
	IG vs. FIR	****	<0.0001	11.3658
	IG vs. SIR	****	<0.0001	11.3874
	IG vs. MR	****	<0.0001	11.8159
Antheraxanthin	IG vs. MG	ns	0.9982	5.7337
	IG vs. B	****	<0.0001	10.1470
	IG vs. FIR	****	<0.0001	10.8273
	IG vs. SIR	****	<0.0001	10.8676
	IG vs. MR	****	<0.0001	10.9353
Violaxanthin	IG vs. MG	ns	0.9942	0.2662
	IG vs. B	****	<0.0001	1.9740
	IG vs. FIR	****	<0.0001	1.9910
	IG vs. SIR	****	<0.0001	2.5025
	IG vs. MR	****	<0.0001	2.4546
Neoxanthin	IG vs. MG	ns	0.0848	0.6591
	IG vs. B	****	<0.0001	1.4565
	IG vs. FIR	ns	0.7879	-0.3987
	IG vs. SIR	ns	0.1099	-1.1451
	IG vs. MR	ns	>0.9999	-0.0602
Capsanthin	IG vs. MG	ns	>0.9999	0.0000
	IG vs. B	*	0.0205	11.3709
	IG vs. FIR	****	<0.0001	12.6744
	IG vs. SIR	****	<0.0001	13.0288
	IG vs. MR	****	<0.0001	13.9654
Capsorubin	IG vs. MG	ns	>0.9999	0.0000
	IG vs. B	ns	>0.9999	0.0000
	IG vs. FIR	ns	0.1374	6.5317
	IG vs. SIR	***	0.0006	7.7201
	IG vs. MR	****	<0.0001	9.7847
Lutein	IG vs. MG	**	0.0021	0.6515
	IG vs. B	*	0.0199	-0.7820
	IG vs. FIR	****	<0.0001	-4.4709
	IG vs. SIR	****	<0.0001	-10.3772
	IG vs. MR	****	<0.0001	-10.3772

	IG vs. MG	**	0.0024	0.6932
	IG vs. B	ns	0.7793	-0.2346
Chlorophyll a	IG vs. FIR	****	<0.0001	-13.4594
	IG vs. SIR	****	<0.0001	-13.4594
	IG vs. MR	****	<0.0001	-13.4594
	IG vs. MG	**	0.0011	0.7103
	IG vs. B	ns	0.5063	0.2557
Chlorophyll b	IG vs. FIR	****	<0.0001	-12.4162
	IG vs. SIR	****	<0.0001	-12.4162
	IG vs. MR	****	<0.0001	-12.4162

[1] The statistical analyses were performed by one way ANOVA and Tukey's multiple comparisons test. [2]*P* value thresholds for different significance levels were 0.05, 0.005, 0.001, and 0.0001, respectively; ns, not significant. [3]Fold changes were calculated with logarithms of the relative pigment contents from different stages plus 0.01 versus IG stage plus 0.01.

Table S3. RNA-seq data quality information.

Sample name	Raw reads	Clean reads	Clean bases	Error rate (%)	Phred-scaled quality		GC content (%)
					Q20 (%)	Q30 (%)	
IG1_1	41429322	40522866	6.07G	0.02	98.23	95.12	42.06
IG1_2	41429322	40522866	6.07G	0.04	93.81	86.01	42.32
IG2_1	38643699	37980229	5.69G	0.02	98.16	94.96	42.40
IG2_2	38643699	37980229	5.69G	0.04	93.26	84.98	42.69
IG3_1	38200860	37581543	5.63G	0.02	98.20	95.02	42.38
IG3_2	38200860	37581543	5.63G	0.04	92.86	84.21	42.71
MG1_1	41212934	40517654	6.07G	0.02	98.16	95.00	41.95
MG1_2	41212934	40517654	6.07G	0.04	93.67	85.72	42.23
MG2_1	37307253	36738204	5.50G	0.02	98.26	95.16	42.29
MG2_2	37307253	36738204	5.50G	0.04	93.42	85.28	42.57
MG3_1	35755956	35161788	5.27G	0.02	98.21	95.06	42.30
MG3_2	35755956	35161788	5.27G	0.04	93.92	86.15	42.57
B1_1	36588097	35935285	5.38G	0.02	98.24	95.13	41.86
B1_2	36588097	35935285	5.38G	0.04	94.30	86.90	42.10
B2_1	37535890	36689474	5.50G	0.02	98.13	94.98	41.42
B2_2	37535890	36689474	5.50G	0.03	94.50	87.38	41.65
B3_1	35414634	34863087	5.22G	0.02	98.27	95.19	41.61
B3_2	35414634	34863087	5.22G	0.04	94.29	86.83	41.84
FIR1_1	34968716	33692856	5.05G	0.02	98.21	95.07	41.52
FIR1_2	34968716	33692856	5.05G	0.04	94.28	86.83	41.73
FIR2_1	37363783	36763467	5.51G	0.02	98.22	95.08	41.36
FIR2_2	37363783	36763467	5.51G	0.04	92.96	84.31	41.68
FIR3_1	37569673	36962379	5.54G	0.02	98.24	95.13	41.55
FIR3_2	37569673	36962379	5.54G	0.04	93.67	85.62	41.83
SIR1_1	40670208	39949755	5.99G	0.02	98.21	95.01	41.67
SIR1_2	40670208	39949755	5.99G	0.03	94.78	87.80	41.77
SIR2_1	39838323	39075085	5.86G	0.02	98.08	94.73	41.72
SIR2_2	39838323	39075085	5.86G	0.04	93.31	84.91	41.89
SIR3_1	35972833	35308766	5.30G	0.02	98.20	94.99	41.59
SIR3_2	35972833	35308766	5.30G	0.04	93.60	85.65	41.76
MR1_1	38020003	37280468	5.59G	0.02	98.23	95.07	41.46
MR1_2	38020003	37280468	5.59G	0.03	94.44	87.09	41.57
MR2_1	43038670	42302749	6.35G	0.02	98.21	95.02	41.66
MR2_2	43038670	42302749	6.35G	0.04	94.24	86.69	41.79
MR3_1	37559601	36826952	5.52G	0.02	98.18	94.95	41.80
MR3_2	37559601	36826952	5.52G	0.04	93.50	85.34	41.95

Table S4. Alignment of the RNA-seq reads and the reference genome.

Sample name	Total reads	Total mapped	Multiple mapped	Uniquely mapped	Read-1	Read-2	Reads map to '+'	Reads map to '-'	Non-splice reads	Splice reads
IG1	81045732	71072830 (87.69%)	2674445 (3.3%)	68398385 (84.39%)	35312214 (43.57%)	33086171 (40.82%)	34221327 (42.22%)	34177058 (42.17%)	46434742 (57.29%)	21963643 (27.1%)
IG2	75960458	65883045 (86.73%)	2091759 (2.75%)	63791286 (83.98%)	33089582 (43.56%)	30701704 (40.42%)	31899055 (41.99%)	31892231 (41.99%)	43557120 (57.34%)	20234166 (26.64%)
IG3	75163086	65400710 (87.01%)	2188915 (2.91%)	63211795 (84.1%)	32871030 (43.73%)	30340765 (40.37%)	31610997 (42.06%)	31600798 (42.04%)	42893684 (57.07%)	20318111 (27.03%)
MG1	81035308	71077097 (87.71%)	2563442 (3.16%)	68513655 (84.55%)	35376511 (43.66%)	33137144 (40.89%)	34265923 (42.29%)	34247732 (42.26%)	47785706 (58.97%)	20727949 (25.58%)
MG2	73476408	64199550 (87.37%)	1944838 (2.65%)	62254712 (84.73%)	32232356 (43.87%)	30022356 (40.86%)	31055290 (42.27%)	31199422 (42.46%)	42193962 (57.43%)	20060750 (27.3%)
MG3	70323576	61830262 (87.92%)	2299568 (3.27%)	59530694 (84.65%)	30686810 (43.64%)	28843884 (41.02%)	29775234 (42.34%)	29755460 (42.31%)	40157651 (57.1%)	19373043 (27.55%)
B1	71870570	63466064 (88.31%)	1843815 (2.57%)	61622249 (85.74%)	31685881 (44.09%)	29936368 (41.65%)	30680763 (42.69%)	30941486 (43.05%)	41693906 (58.01%)	19928343 (27.73%)
B2	73378948	64759924 (88.25%)	2069770 (2.82%)	62690154 (85.43%)	32167426 (43.84%)	30522728 (41.6%)	31256571 (42.6%)	31433583 (42.84%)	44762370 (61%)	17927784 (24.43%)
B3	69726174	61846454 (88.7%)	1855616 (2.66%)	59990838 (86.04%)	30853348 (44.25%)	29137490 (41.79%)	29920979 (42.91%)	30069859 (43.13%)	40126444 (57.55%)	19864394 (28.49%)
FIR1	67385712	53208533 (78.96%)	1547050 (2.3%)	51661483 (76.67%)	26593435 (39.46%)	25068048 (37.2%)	25729076 (38.18%)	25932407 (38.48%)	34682917 (51.47%)	16978566 (25.2%)
FIR2	73526934	64409262 (87.6%)	1789097 (2.43%)	62620165 (85.17%)	32509732 (44.21%)	30110433 (40.95%)	31188328 (42.42%)	31431837 (42.75%)	42106836 (57.27%)	20513329 (27.9%)
FIR3	73924758	64669947 (87.48%)	1908417 (2.58%)	62761530 (84.9%)	32421100 (43.86%)	30340430 (41.04%)	31244790 (42.27%)	31516740 (42.63%)	43069425 (58.26%)	19692105 (26.64%)
SIR1	79899510	70857393 (88.68%)	2239174 (2.8%)	68618219 (85.88%)	35084645 (43.91%)	33533574 (41.97%)	34164021 (42.76%)	34454198 (43.12%)	46920729 (58.72%)	21697490 (27.16%)
SIR2	78150170	68719642 (87.93%)	2203834 (2.82%)	66515808 (85.11%)	34329055 (43.93%)	32186753 (41.19%)	33102530 (42.36%)	33413278 (42.76%)	46653355 (59.7%)	19862453 (25.42%)
SIR3	70617532	61921843 (87.69%)	1946772 (2.76%)	59975071 (84.93%)	30930402 (43.8%)	29044669 (41.13%)	29868722 (42.3%)	30106349 (42.63%)	41682089 (59.03%)	18292982 (25.9%)
MR1	74560936	65839810 (88.3%)	2119865 (2.84%)	63719945 (85.46%)	32653979 (43.8%)	31065966 (41.67%)	31771274 (42.61%)	31948671 (42.85%)	43786017 (58.73%)	19933928 (26.74%)
MR2	84605498	74932528 (88.57%)	2597500 (3.07%)	72335028 (85.5%)	37104437 (43.86%)	35230591 (41.64%)	36076273 (42.64%)	36258755 (42.86%)	50438546 (59.62%)	21896482 (25.88%)
MR3	73653904	64745079 (87.9%)	2252133 (3.06%)	62492946 (84.85%)	32229107 (43.76%)	30263839 (41.09%)	31182021 (42.34%)	31310925 (42.51%)	42732408 (58.02%)	19760538 (26.83%)

Table S5. Genes for carotenoid biosynthesis enzymes identified from the chili pepper *Zunla-1* genome.

Accession number	Name
Capana00g002450	geranylgeranyl diphosphate synthase 1 (GGPPS1)
Capana00g002451	geranylgeranyl diphosphate synthase 2 (GGPPS2)
Capana00g002452	geranylgeranyl diphosphate synthase 3 (GGPPS3)
Capana03g002322	geranylgeranyl diphosphate synthase 4 (GGPPS4)
Capana04g000412	geranylgeranyl diphosphate synthase 5 (GGPPS5)
Capana05g000800	geranylgeranyl diphosphate synthase 6 (GGPPS6)
Capana00g004199	GGPPS small subunit, type I (SSUI)
Capana09g002331	GGPPS small subunit, type II (SSUII)
Capana01g001510	phytoene synthase 1 (PSY1)
Capana02g002284	phytoene synthase 2 (PSY2)
Capana04g002519	phytoene synthase 3 (PSY3)
Capana10g000838	phytoene synthase 4 (PSY4)
Capana03g000054	15-*cis*-phytoene desaturase (PDS)
Capana12g000229	15-*cis*-ζ-carotene isomerase (ZISO)
Capana08g001314	ζ-carotene desaturase 1 (ZDS1)
Capana08g001316	ζ-carotene desaturase 2 (ZDS2)
Capana00g004805	carotene isomerase 1 (CRTISO1)
Capana11g002179	carotene isomerase 2 (CRTISO2)
Capana05g000023	lycopene β-cyclase 1 (LCYB1)
Capana10g002320	lycopene β-cyclase 2 (LCYB2)
Capana00g002014	lycopene ε-cyclase 1 (LCYE1)
Capana07g001071	lycopene ε-cyclase 2 (LCYE2)
Capana09g000177	lycopene ε-cyclase 3 (LCYE3)
Capana12g001558	lycopene ε-cyclase 4 (LCYE4)
Capana12g001560	lycopene ε-cyclase 5 (LCYE5)
Capana12g001589	lycopene ε-cyclase 6 (LCYE6)
Capana01g002976	carotene ε-hydroxylase 1 (CYP97C1)
Capana05g002138	carotene ε-hydroxylase 2 (CYP97C2)
Capana06g001091	carotene ε-hydroxylase 3 (CYP97C3)
Capana10g001912	carotene ε-hydroxylase 4 (CYP97C3)
Capana12g001743	carotene β-hydroxylase (CYP97A)
Capana03g002170	β-carotene hydroxylase 1 (BCH1)
Capana06g002492	β-carotene hydroxylase 2 (BCH2)
Capana02g003105	zeaxanthin epoxidase 1 (ZEP1
Capana06g001608	zeaxanthin epoxidase 2 (ZEP2)
Capana12g001449	violaxanthin de-epoxidase 1 (VDE1)
Capana12g001844	violaxanthin de-epoxidase 2 (VDE2)
Capana06g000615	capsanthin/capsorubin synthase (CCS)

Gene sequences are accessible through the *Zunla-1* genome (https://solgenomics.net/tools/blast/, accessed on 1 January 2019).
